# Isolation and partial characterization of *Salmonella* Gallinarum bacteriophage

**DOI:** 10.1016/j.sjbs.2022.02.007

**Published:** 2022-02-14

**Authors:** Fawzi Al-Razem, Hiba Al-Aloul, Murad Ishnaiwer, Razan Qadi

**Affiliations:** a College of Medicine and Health Sciences, Palestine Polytechnic University, P.O. Box 198 Hebron, Palestine; b Medical Microbiology, MiHAR Lab, University of Nantes, 44200 Nantes, France; c Applied Biology Program, College of Applied Sciences, Palestine Polytechnic University, P.O. Box 198 Hebron, Palestine

**Keywords:** *Salmonella* Gallinarum, Bacteriophage, Poultry, Phage therapy, Antibiotic resistance

## Abstract

Infections caused by *Salmonella* remain a major public health problem worldwide. Animal food products, including poultry meat and eggs, are considered essential components in the individual’s daily nutrition. However, chicken continues to be the main reservoir for *Salmonella* spp.

Poultry farmers use several types of antibiotics to treat pathogens. This can pose a health risk as pathogens can build antibiotic resistance in addition to the possibility of accumulation of these antibiotics in food products. The use of phages in treating poultry pathogens is increasing worldwide due to its potential use as an effective alternative to antibiotics. Phages have several advantages over antibiotics; phages are very specific to target bacteria, less chances of developing secondary infections, and they only replicate at the site of infection.

Here we report the isolation of a bacteriophage from chicken feces. The isolated bacteriophage hosts on *Salmonella* Gallinarum, a common zoonotic infection that causes fowl typhoid, known to cause major losses to poultry sector. The isolated bacteriophage was partially characterized as a DNA virus resistant to RNase digestion with approximately 20 Kb genome. SDS-PAGE analysis of total viral proteins showed at least five major bands (21, 28, 42, 55 and 68 kDa), indicating that this virus is relatively small compared to other known poultry phages. The isolated bacteriophage has the potential to be an alternative to antibiotics and possibly reducing antibiotic resistance in poultry farms.

## 1 Introduction

Zoonotic diseases caused by Salmonella spp. are still a major concern for public health, in addition to their impact on farmers and livestock sector in general (Atterbury et al., 2007). Fowl typhoid is an acute septicaemic disease caused by *Salmonella enterica* serovar Gallinarum (*Salmonella* Gallinarum) that infect poultry. It is transmitted through fecal-oral contact and respiratory routes and the spread of this disease in infected poultry farms is usually difficult to control (Shivaprasad, 2000). It can easily spread within days if appropriate farm management is not adhered to. Poor drainage systems, cleanness, air, water, feed and vectors such as humans, rodents and insects contribute to cross infections that result in increased morbidity and mortality rates in poultry farms (Atterbury et al., 2007).

The disease causes high mortality in chicken particularly during the first two weeks of age if not diagnosed. In older flocks, the clinical signs of fowl typhoid include dehydration, diarrhea, anemia, depression, laboured breathing, decreased egg production and hatchability. Pathological changes appear in the subacute and chronic stages with small lesions, severe swelling, and discoloration in liver and spleen (Kwon et al., 2010, Hong et al., 2013, Fakhouri et al., 2019).

Poultry can be infected with several other diseases caused by a diversity of viral, bacterial and fungal pathogens. Antiviral and antifungal agents are rarely used in poultry as viruses and fungal diseases are usually controlled by vaccination. Bacterial diseases are controlled through the use of dozens of different antibacterial chemicals that have become common in livestock farms throughout much of the twentieth century (Page and Gautier, 2012, Fakhouri et al., 2019).

Antibiotics are still the main antimicrobial treatments used to treat fowl typhoid, even though only partial success have been reached in eliminating this farm pathogen (Olowe et al., 2007, Munita et al., 2016, Parvej et al., 2016, Penha et al., 2016). The misuse of antibiotics has resulted in the development of resistant bacteria to many commonly used antibiotics. The misuse of antibiotics in farms can lead not only to the possibility of generating multidrug resistance bacteria, but also can pose a health risk when retained in the poultry food products (Munita et al., 2016, Prajapati et al., 2018, Seo et al., 2019; Sánchez‐Salazar et al., 2020). Several studies reported a multidrug resistant *Salmonella*, particularly against tetracycline, ampicillin, kanamycin, sulfadiazine, and chloramphenicol and quinolones (Tang et al., 2019, Xiong et al., 2020).

It is highly possible that the inappropriate use of antibiotics in some animal husbandries may threaten their efficacy as well since antibiotics are also used to treat human pathogens (Page and Gautier, 2012, Fakhouri et al., 2019, Sánchez-Salazar et al., 2020). For example, ceftriaxone antibiotic is used to treat *Salmonella* infections in children. The efficacy of this antibiotic was reduced when used to treat ceftriaxone-resistant *Salmonella* in a child and several other ceftriaxone-resistance cases were reported worldwide (Fey et al., 2000, White et al., 2001, Al kraiem et al., 2019). To add to this problem, it is suggested that nearly 75% of all antibiotics used in animal farms are not fully absorbed by the animal tissues, but rather pass to the environment. This will expose these antibiotics to new bacteria and possibly creating new resistant strains (Chee-Sanford et al., 2009, Huang et al., 2018, Manyi-Loh et al., 2018). In searching for a replacement to antibiotics, phage therapy was introduced as a possible alternative to antibiotics. It is thought to have several advantages over antibiotics, including the high host specificity and auto-dosing (Morrisette et al., 2019). Phage therapy focus on the isolation of new bacteriophages that can be efficient killers of bacteria. It is possible that some bacteria will still show natural resistance to bacteriophages, but taking into account the fast evolution of viruses, it is expected that many mutants of phages exist for each bacterial pathogens. Using phage mixtures and rotations can also limit the development of resistant bacterial mutants (Nilsson, 2019, Sommer et al., 2019).

The overall objective of this study was to isolate a bacteriophage that can host on the fowl typhoid pathogen caused by *S.* Gallinarum and determine if the isolated phage is capable of lysing this pathogenic bacterium.

## 2 Materials and methods

### 2.1 Sample collection, culture media, and growth conditions

*Salmonella* Gallinarum isolates were obtained from the Palestine-Korea Biotechnology Research Center, Hebron, Palestine**.** Biosecurity and institutional biosafety have been adhered to. The culture of *Salmonella* bacteria was obtained by mixing 100 µl of *S.* Gallinarum with 900 µl Luria-Bertani broth (LB) (5 g Tryptone, 5 g NaCl, 2.5 g Yeast extract in 500 ml H_2_O) and incubated overnight at 37 °C with constant shaking at 180 rpm.

### 2.2 Phage isolation

Samples of poultry feces were obtained from local chicken farms in Hebron city. To search for the presence of phages, 2 g of feces were incubated in 13 ml LB-broth for 9 h at 37 °C in the shaker incubator set at 180 rpm. To separate phage from bacteria, 100 µl of chloroform were added to the samples, vortexed for 2 min and centrifuged at 6000 rpm for 20 min at room temperature. The collected supernatant was then transferred to a new microfuge tube and filtered through 0.45-µm Cellulose Acetate filter to remove debris.

For the isolation of *S.* Gallinarum specific phages*,* a protocol from Fakhouri et al., 2019 was followed with modifications as follows. Two ml of the above filtrate were mixed with 12 ml of LB-broth, 1 ml of an overnight *S.* Gallinarum culture and incubated in shaker incubator set at 160-rpm for 7 h at 37 °C. For further enrichment, chloroform was added as above and the culture was then centrifuged at 6000 rpm for 15 min. The supernatant transferred to falcon tube with 1 ml of fresh *S.* Gallinarum pure culture and the volume was completed to 25 ml of LB-broth, followed by overnight incubation at 37 °C in shaker incubator. Chloroform was then added as above, vortexed, and the culture was centrifuged at 13000 rpm for 15 min. The supernatant was filtrated through 0.20-µm filter and phage filtrate stored at 4 °C until further use.

### 2.3 Bacteriophage lysis testing

Phage lysis was carried out as described by Fakhouri et al. (2019). Briefly, an overnight *S.* Gallinarum pure culture was prepared. To identify host-specificity of the phages in the above mixture, 200 μl from the *S.* Gallinarum culture spread on two SS-Agar plates, one of which was used as a control and 5 drops of 10 μl from the phage mixture pippeted on the other plate before both plates were incubated overnight at 37 °C. The clear lysed area was then cut and placed in a tube containing broth LB media, incubated overnight and phage was isolated as above.

Lysis efficiency of phages isolated from the previous step were tested again into two trials. The first trial was done by pipetting 3 drops (7 µl) from phage filtrate on newly prepared *S.* Gallinarum LB separated agar plate, the second trial spotted 7 µl from the phage filtrate on specific site on plate by pipetting 5 drops (7 µl) from phage filtrate on newly prepared *S.* Gallinarum LB agar plates. These steps were done to confirm the phage ability to lyse its *S.* Gallinarum host in a reproducible manner. Phage lysis was monitored for two days.

### 2.4 Bacteriophage genome isolation

Bacteriophage genome was extracted according to a protocol by Ishnaiwer and Al-Razem (2013) with minor modifications as follows. A 1000 µl of saturated ammonium sulfate containing 1 µl of 2-mercaptoethanol were mixed with the phage sample (1000 µl) for 5 min. After centrifuging at 15,000 rpm for 8 min at 4 °C, the supernatant was removed and the pellet was dissolved in 200 µl 1% SDS and 200 µl 0.5 N NaOH. The dissolved pellet was then centrifuged at 13,000 rpm for 5 min. To the clear supernatant, 400 µl of 3 N Sodium Acetate were added in addition to 420 µl- 0.6 vol of isopropanol to precipitate the genome and allowed to incubate for 15 min at room temperature. The mixture was then centrifuged at 15,000 rpm for 15 min and the resulting pellet incubated with 700 µl of 100 µg/µl of proteinase K at 37 °C for 30 min. Finally, the phage genome was precipitated using 70% ethanol and the pellet collected in 200 µl TE buffer (100 ml TrisCl, 200 ml of 0.5 M EDTA pH 8.0 added to 880 ml ddH_2_O) after the 2 min centrifugation at 8,000 rpm.

The extracted phage genome was detected by running 5 µl of the sample mixed with µl 6X loading dye (25% (v/v) Ficoll, 1% (w/v) Orange G, 0.5% (w/v) Bromphenolblue and 0.5% (w/v) XylenCyanol) and 15 µl distilled water before loading on 1X TBE buffer (242 g Tris, 57 g boric acid ,0.5 M EDTA pH 8.0 to 1L) agarose gel electrophoresis. The Separation was carried out in 1X TBE buffer at 80 V for 30 min and documented using a gel documentation system.

### 2.5 Dnase and RNase treatment of phage genome

The nature of phage genome was determined by treating the purified genome with DNAse and RNAse. 10 µl of genome sample was incubated with 3 µl of DNase and the same with RNase for 35 min at 37 °C. The mixtures were then loaded on 0.5% agarose gel and the undigested genome was used as a control.

### 2.6 SDS-PAGE analysis

Total phage proteins were separated on a 10% SDS-PAGE, stained with 0.1% (w/v) Coomassie Brilliant Blue R-25. A 5% stacking gel and 8% resolving gel were prepared as described by Fakhouri et al. (2019). Samples of total phage proteins were prepared by mixing 100 µl of each phage samples with 100 µl 5x SDS Gel-loading buffer (250 mMTris- Cl (pH 8.6), 10% (w/v) SDS, 0.02% (w/v) bromophenol blue, 30% (v/v) glycerol and 5% β-mercaptoethanol) and then heated for 3 min at 100 °C. Following sample loading, the gel electrophoresis was allowed to run first at 80 V for 1 h until the protein passed the stacking gel, then increased to 150 V until the run completed. The gel was then placed in Coomassie Brilliant Blue stain solution (0.3 g of Coomassie Brilliant Blue powder in 90 ml of methanol, glacial acid solution (5 methanol: 4 water: 9 glacial acetic acid) for 3 h with continuous shaking. Following staining, the gel was transferred to destaining solution and fixing solution (1 glacial acetic acid: 2 methanol: 7 water) for 1–2 h. The destaining solution was renewed twice each 15 min.

## 3 Results

### 3.1 Isolated bacteriophages filtrate

**Fig. 1:**
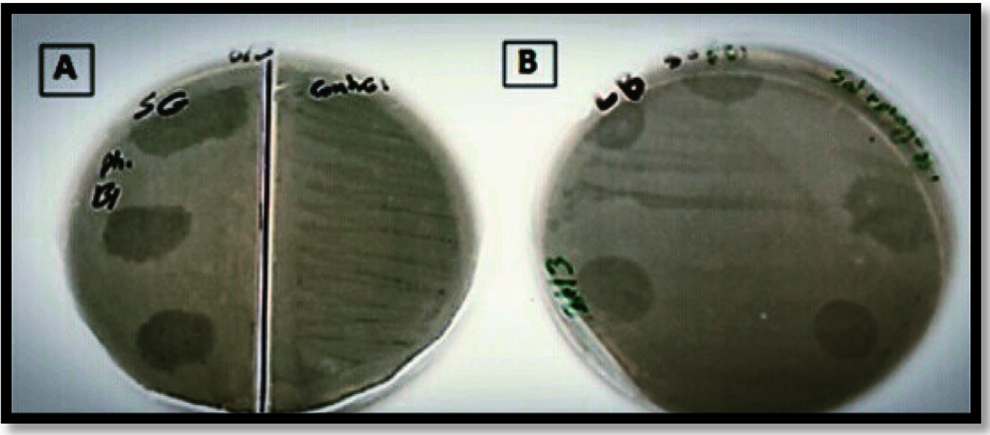
*Salmonella* Gallinarum cultures on LB-agar plates showing bacteriophage lysis detected after 24 hr incubation. a: Clear round plaques appeared after 7 µl phage filtrate isolated from the first purification trial was spotted on *Salmonella* Gallinarum cultures. b: Clear round plaques appeared after phage filtrate isolated from the second purification trial was spotted as above.

In the first purification trial, three clear round plaques, each with approximately 1 cm in diameter appeared after 24 hr incubation at 37 °C (Fig. 1a). Five clear round plaques, each also with 1 cm diameter appeared in the second trial (Fig. 1b). Each plaque formed after 7 µl from the phage filtrate were spotted on the plate surface.

### 3.2 Agarose analysis of the isolated bacteriophage

**Fig. 2:**
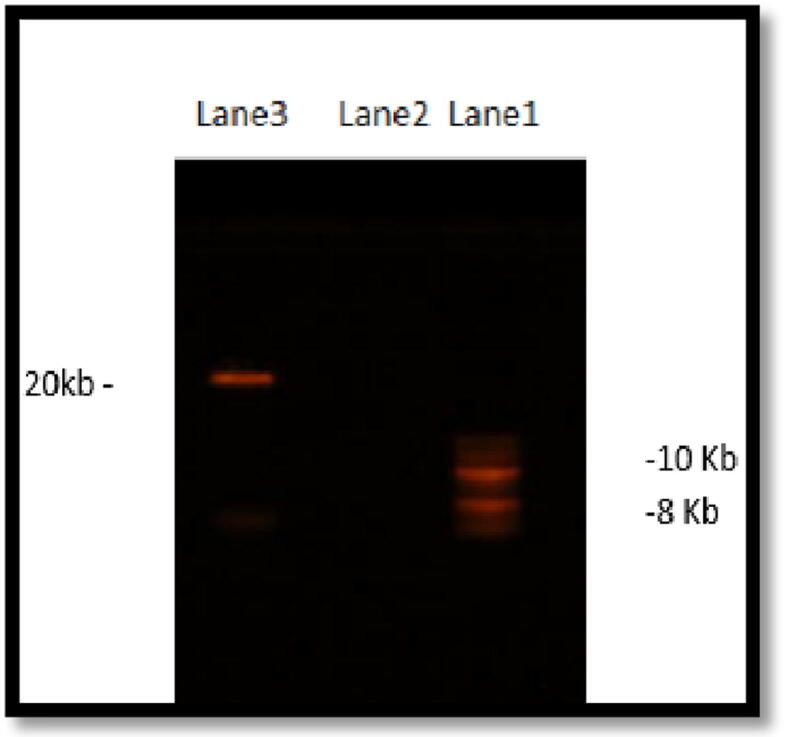
Genome size estimation of the isolated bacteriophage. Bacteriophage sample was prepared by mixing 5 µl of purified phage genome, added to it 5 µl of (6X) loading dye and (10 µl) of distilled H_2_O and loaded on 0.5% agarose gel electrophoresis. Lane 1 contained 10 kb ladder and Lane 2 contained 10 µl of H_2_O and 15 µl of the loading dye. Lane 3 contained the bacteriophage sample. As shown the band of the genome was detected and estimated to be approximately 20 kb on a gel documentation system.

The estimated size of the isolated phage genome was determined using agarose gel and a high molecular weight 10 Kb DNA ladder. Isolated phage genome mixed was loaded on a 0.5% agarose gel and allowed to run until fully resolved (Fig. 2). Following a 35 min run, a band appeared and estimated to be approximately 20 Kb in size.

### 3.3 Characterization of the isolated bacteriophage

**Fig. 3:**
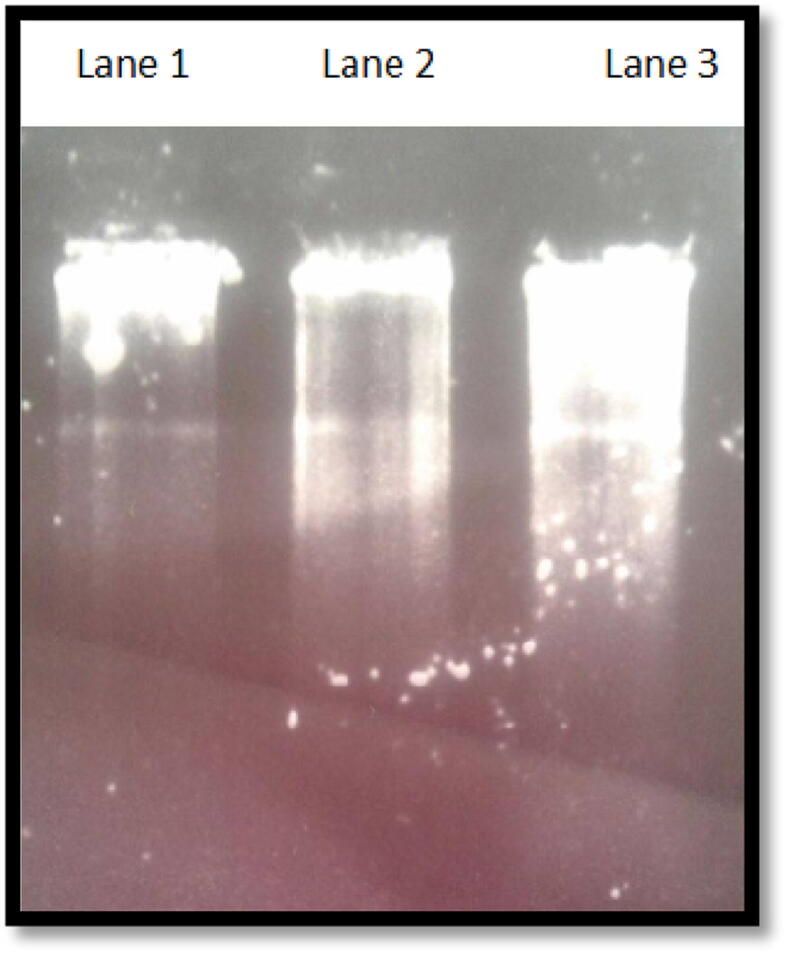
The isolated bacteriophage has a DNA genome. Lane 1 contained the undigested genome. Lane 2 contained genome sample treated with (RNase). Lane 3 contained bacteriophage genome treated with (DNase). All reactions loaded on a (0.5 %) agarose gel. As shown, the genome has resisted RNase digestion, whereas it smeared by the DNase, an indication of possible DNA composition.

To determine the nucleic acid composition of the isolated phage genome, nuclease treatment using DNase and RNase was carried out on isolated phage genome. After bacteriophage genome nuclease treatment, the mixtures were mixed with loading dye and loaded on a 0.5% agarose gel. Agarose gel analysis of the digested genomes revealed that the phage genome was likely a DNA genome as it was more sensitive to DNase digestion and resistant to RNase (Fig. 3)

### 3.4 SDS-PAGE analysis of the isolated bacteriophage

**Fig. 4:**
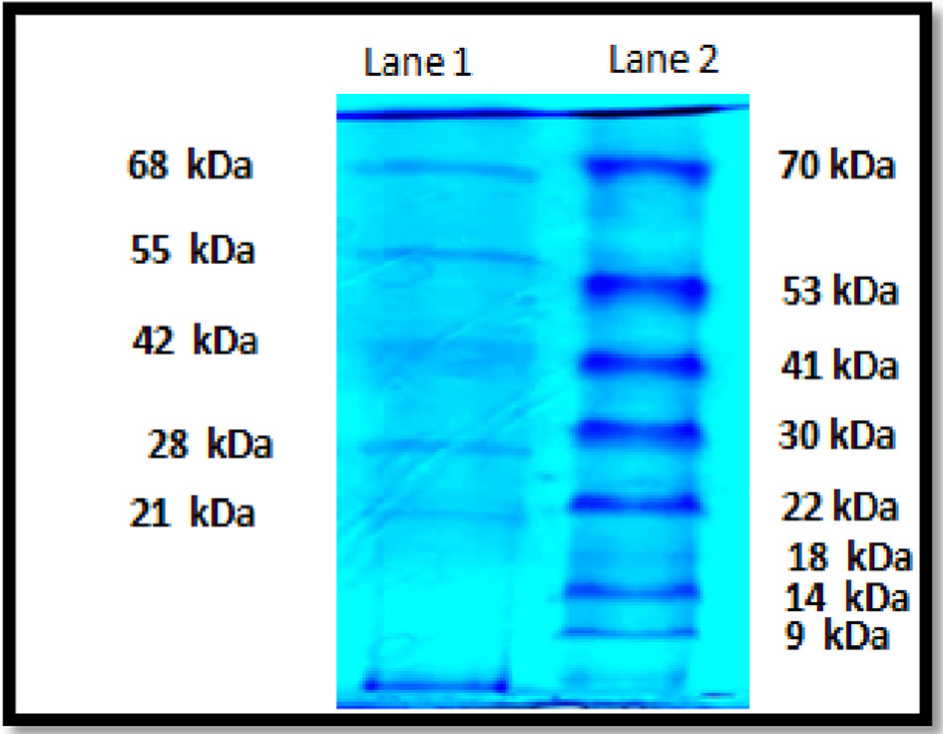
SDS-PAGE gel analysis of phage proteins. Lane 1 shows five bands and Lane 2 contains high molecular weight protein ladder. As shown, at least five major bands (21, 28, 42, 55 and 68 kDa) were detected.

The phage total proteins were analyzed on SDS-PAGE. Samples were electrophoresed on a 10% SDS-PAGE and stained with Coomassie brilliant blue. Stained gel showed five distinct protein bands with estimated sizes of approximately (21 kDa, 28 kDa, 42 kDa, 55 kDa, and 68 kDa) **(**Fig. 4**)**.

## 4 Discussion

Poultry diseases associated with bacterial infections are considered serious problems and usually result in significant economic losses. These infections are dominated by *Salmonella* and avian *Escherichia coli* pathogens in addition to other microbes that differ in scale from place to place. *Salmonella* infections are widespread and are sometimes difficult to control, leading many poultry farmers to use extensive antibiotic treatments. The use of these antibiotics has contributed to the multidrug resistance seen in several *Salmonella* strains, few of which have emerged with clinical implications (Ajiboye et al., 2009).

Several isolated bacteriophages have been used in phage therapy against *Salmonella* and appeared to have promising potential as efficient treatments that could replace antibiotics (Tang et al., 2019, Żbikowska et al., 2020). It is very interesting to know that the first trial of phage therapy against *S.* Gallinarum in chicken was done by D’Herelle in 1919. This followed by the use of phage therapy in humans in 1925 when D’Herelle used isolated phage to treat four cases of bubonic plague by direct injection of phage into the buboes (Atterbury, 2006, Anisimov and Amoako, 2006, Belay et al., 2018). Additionally, phages (ST4, L13, and SG3) against *S.* Gallinarum infection were isolated and their efficiency against *Salmonella* was tested in vivo (Hong et al., 2013), another proof of the possibility of using bacteriophage in phage therapy against pathogens affecting humans directly or indirectly.

The genome of the bacteriophage isolated in this study was analyzed using 0.5% agarose gel electrophoresis. A DNA band of approximately 20 kb (Fig. 2) was visible and was in the range of other bacteriophage genomes reported for *Salmonella* by Ngangbam and Devi (2012) and slightly bigger than avian *E. coli* bacteriophage genome which was estimated to approximately 17 kb (Fakhouri et al., 2019). DNase treatment of the isolated bacteriophage indicated that it is a DNA virus (Fig. 3). This is consistent with other bacteriophages isolated against avian pathogens, including *Salmonella* and *E. coli*. It has been reported that bacteriophages that infected *Salmonella* species are classified as Podoviridae, short-tailed phages, and are double stranded DNA (Ngangbam and Devi, 2012). For further characterization of the isolated bacteriophage, total phage proteins were extracted and phage binding proteins were disrupted by the addition of NaOH and SDS and proteins precipitated by the addition of sodium acetate in the presence of β-mercaptoethanol. Five major protein bands with molecular weights of approximately 21, 28, 42, 55 and 68 kDa were visible on SDS-PAGE. It is likely that the phage genome encodes for other capsid and/or DNA-binding proteins, even though no minor structural protein appears. However, the protein profile is not identical to that reported by Ngangbam and Devi (2012) for *Salmonella* phages isolated from natural environment. This further emphasizes the high variations observed in bacteriophages.

Antibiotic resistance in bacteria has become a major threat for public health at a global scale, and these isolated bacteriophages may open the door for new opportunities to control several pathogens by phage therapy. Bacteriophages represent an effective way to kill pathogenic bacteria and can be used as potential alternative for antibiotics due to their ability to kill specific types of pathogenic bacteria. Phages have normally no negative effects on the normal flora and usually leave the chicken after killing the pathogenic bacteria. The isolated bacteriophage show high effectiveness against *S.* Gallinarum that causes typhoid fever and results in major losses for poultry farmers in addition to the possibility of transformation to farmers and workers in the poultry farms. Future in vivo and farm studies will be prove its efficiency in treating *Salmonella* infections.

We believe this bacteriophage may be an effective alternative to antibiotics and a more cost-effective treatment against the pathogenic *Salmonella.*

## 5 Conclusion

A significant increase in antibiotic resistance among bacteria is observed worldwide. The use of phages in treating poultry pathogens is increasing because its potential use as an effective alternative to antibiotics. The isolated bacteriophage is expected to be a better and safe method of treating one of the major pathogens affecting poultry and could result in a significant increase in poultry production and safety. Phage therapy could therefore become an efficient alternative to antibiotics as it is proved to be safe and environment friendly.

## 6 Ethics approval

Not applicable.

## 7 Consent to participate

All authors consent to participate in this manuscript.

## 8 Consent for publication

All authors consent to publish this manuscript in Saudi Journal of Biological Science.

## 9 Availability of data and material

Data will be available on request to the corresponding and second author.

## Declaration of Competing Interest

The authors declare that they have no known competing financial interests or personal relationships that could have appeared to influence the work reported in this paper.
